# Acinetobacter uliginosus sp. nov. and Acinetobacter halobius sp. nov. isolated from soil

**DOI:** 10.1099/ijsem.0.007192

**Published:** 2026-06-04

**Authors:** Cong-Guo Ran, Feng-Lan Liu, Tong Wu, Rashidin Abdugheni, Nan Zhou, Shuang-Jiang Liu

**Affiliations:** 1 State Key Laboratory of Microbial Diversity and Innovative Utilization and Environmental Microbiology Research Center at Institute of Microbiology, Chinese Academy of Sciences, Beijing, 100101, PR China; 2 University of the Chinese Academy of Sciences, Beijing, 100049, PR China; 3 State Key Laboratory of Microbial Biotechnology, Shandong University, Qingdao, 266237, PR China; 4 Department of Microbiology, School of Basic Medical Sciences, Xinjiang Medical University, Urumqi 830017, PR China

**Keywords:** *Acinetobacter*, *Moraxellaceae*, phylogenomics, polyphasic taxonomy

## Abstract

Two bacterial strains, designated BSP-53^T^ and BSP-153^T^, were isolated from soil samples collected from Beijing Olympic Forest Park, China. Phylogenomic reconstruction based on core genes robustly positioned the strains as two distinct evolutionary lineages within the genus *Acinetobacter*. Both strains were Gram-negative, aerobic, non-motile and short rods. Their major cellular fatty acids consisted of summed feature 3 (C_16 : 1_* ω*7*c*/C_16 : 1_* ω*6*c*), C_18 : 1_* ω*9*c*, C_16 : 0_, C_12 : 0_ and C_12 : 0_ 3-OH. The predominant polar lipids included diphosphatidylglycerol, phosphatidylethanolamine and phosphatidylglycerol. The average nucleotide identity values between the novel strains and their closest *Acinetobacter* type strains were below 95%, with corresponding digital DNA–DNA hybridization values below 70%. Based on polyphasic taxonomic characterization, including chemotaxonomic, phenotypic, phylogenetic and genomic analysis, we propose that strains BSP-53^T^ and BSP-153^T^ represent two novel species of the genus *Acinetobacter*, for which the names *Acinetobacter uliginosus* sp. nov. and *Acinetobacter halobius* sp. nov. are designated. The type strains are BSP-53^T^ (=CGMCC 1.62070^T^=KCTC 8190^T^) and BSP-153^T^ (=CGMCC 1.62071^T^=KCTC 8191^T^), respectively.

## Introduction

The genus *Acinetobacter*, a member of the family *Moraxellaceae*, was originally established by Brisou and Prévot [1], with its species diversity continually expanding [2,3]. *Acinetobacter lwoffii* and *Acinetobacter calcoaceticus* were the first two species identified. As of the date of this communication, the genus *Acinetobacter* includes 87 validly published and correct names (https://lpsn.dsmz.de//genus/acinetobacter). The genus *Acinetobacter* represents a physiologically and metabolically diverse group, exhibiting considerable biochemical and genetic heterogeneity. *Acinetobacter* spp. are ubiquitous in nature and have been isolated from a wide range of sources, including humans, animals, plants, water and soil [4]. Some species have been isolated from more specific habitats, such as floral nectar, elephant faeces, *Populus* bark and lead-zinc ore [5,8]. All members of the genus are Gram-negative, non-motile, oxidase-negative, non-haemolytic and aerobic bacteria [9].

The genus *Acinetobacter* has emerged as a notable example of microbial adaptability, demonstrating exceptional capabilities in environmental persistence and evolution of antimicrobial resistance [10,11], making it a critical focus of microbiological research. However, beyond its biological interest, the genus poses a growing public health concern. Of particular note, multidrug-resistant strains readily spread across clinical and environmental settings, exemplifying a serious One Health challenge [12]. Zoonotic transmission of *Acinetobacter baumannii* clones and emerging threats from *Acinetobacter bereziniae* and *Acinetobacter junii* further underscore this issue [13,16]. This study contributes to this field by characterizing two novel strains isolated from soil, which exhibit distinct taxonomic and phenotypic traits.

## Isolation and ecology

Soil samples were collected from the riparian zone of the Longxing (dragon-shaped) aquatic system in Beijing Olympic Park, China (39° 59′ 20.72″ N 116° 23′ 51.63″ E), a site previously studied for microbial diversity and ecology [17,19]. Approximately 50.0 g of soil was aseptically transferred to a sterile bag, transported on ice and homogenized by vortexing for 1 h in 100 ml of sterile PBS (pH 7.2). Serial tenfold dilutions (10⁻¹–10⁻⁷) were prepared in sterile PBS. Aliquots (200 µl) of each dilution were spread onto Reasoner's 2A (R2A) agar plates and incubated at 30 °C for 7 days. R2A medium was chosen for its ability to support a wide spectrum of environmental micro-organisms, including slow-growing and oligotrophic bacteria [20]. Among 200 purified isolates, two strains, BSP-53^T^ and BSP-153^T^, exhibited 16S rRNA gene sequence similarities below the 98.7% [21] threshold for species delineation when compared to known type strains using the EzBioCloud (https://www.ezbiocloud.net/) [22,24], indicating their potential as novel taxa. The strains were preserved as 20% (v/v) glycerol suspensions at −80 °C and deposited in the China General Microbiological Culture Collection Centre (CGMCC) and the Korean Collection for Type Cultures (KCTC) under accession numbers BSP-53^T^ (=CGMCC 1.62070^T^=KCTC 8190^T^) and BSP-153^T^ (=CGMCC 1.62071^T^=KCTC 8191^T^).

## Phylogeny and genome features

The 16S rRNA genes were amplified from pure cultures with universal primers 27F (5′-AGAGTTTGATCCTGGCTCAG-3′) and 1492R (5′-GGTTACCTTGTTACGACTT-3′). The obtained 16S rRNA gene sequences were aligned with those of phylogenetically related type strains retrieved from the EzBioCloud database. Additionally, comprehensive searches were conducted in public sequence repositories (including GenBank, EMBL and DDBJ databases) to identify and incorporate similar sequences for comparative analysis. Phylogenetic trees were constructed using the neighbour-joining (NJ) method [25] based on Kimura’s two-parameter model [26], the maximum-likelihood (ML) method [27] based on the Tamura–Nei model and the maximum parsimony (MP) method in mega 7.0 [28], with bootstrap values based on 1,000 replications.

The 16S rRNA gene sequences of strains BSP-53^T^ and BSP-153^T^ showed the highest similarities of 98.22 and 98.52% to the type strains of *Acinetobacter kookii* ANC 4667^T^ [29] and *Acinetobacter tibetensis* Y-23^T^ [30], respectively. These values are below the 98.7% threshold recommended for the delineation of a novel species. Phylogenetic analysis based on the 16S rRNA gene placed both BSP-53^T^ and BSP-153^T^ within the genus *Acinetobacter*, while revealing that each formed a distinct and separate lineage in the NJ (Fig. S1, available in the online Supplementary Material), ML (Fig. S2) and MP (Fig. S3) trees. This consistent phylogenetic topology, supported by the low 16S rRNA gene sequence similarities, strongly indicates that strains BSP-53^T^ and BSP-153^T^ represent two novel species of the genus *Acinetobacter*.

Genomic DNA of strains BSP-53^T^ and BSP-153^T^ was extracted following a previously described method [31]. Sequencing libraries were constructed with the Illumina TruSeq DNA Library Preparation Kit. Briefly, the DNA was sheared to fragments of 300–500 bp using a Covaris M220 ultrasonicator, followed by end repair and adapter ligation. Libraries with insert sizes of 350–550 bp were selected using AMPure XP beads and then sequenced on an Illumina HiSeq X Ten platform to generate 2×150 bp paired-end reads. The raw sequencing data were subjected to quality control and adapter trimming with Fastp (v0.20.0). The raw sequence data were quality assessed, trimmed and assembled using SOAPdenovo (v2.04) [32], and the assembly quality was assessed as described previously [33]. Gene prediction and annotation were carried out using Prodigal (v2.6.3) [34] and GeneMarkS (v4.3) [35]. Genome-based taxonomic classification was performed using the Genome Taxonomy Database Toolkit (GTDB-TK) [36]. For taxonomic delineation, the average nucleotide identity (ANI) was calculated using the EzBioCloud ANI calculator, while digital DNA–DNA hybridization (dDDH) values were estimated with the Genome-to-Genome Distance Calculator (GGDC 2.1) [37].

**Fig. 1.**
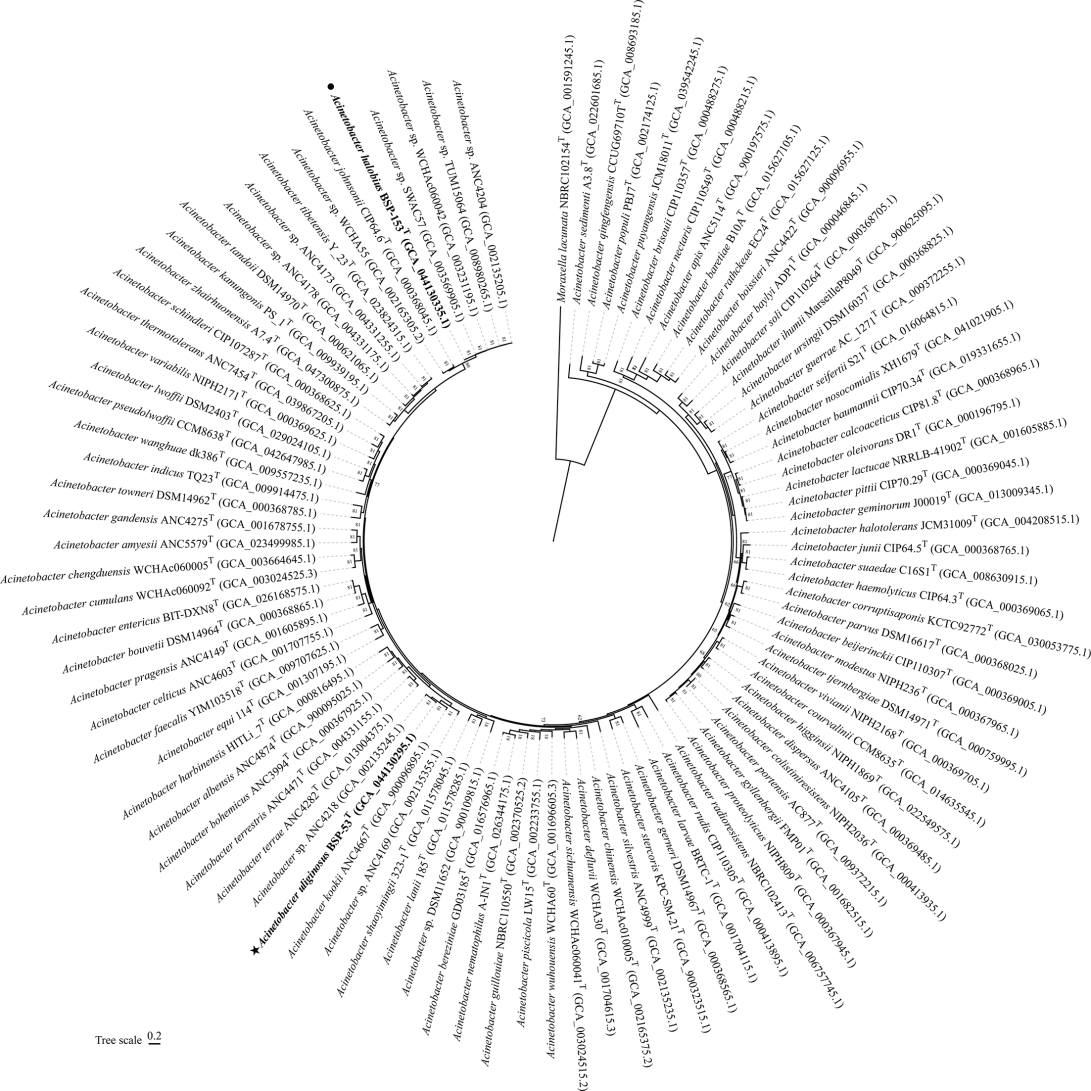
Phylogenomic trees of strains BSP-53^T^ and BSP-153^T^ among the members of the genus *Acinetobacter*. Phylogenetic relationships at the genomic level of strains BSP-53^T^ and BSP-153^T^ and the species of the genus *Acinetobacter*. Phylogenomic tree of each strain and its closely related strains based on 81 bacterial core gene sequences constructed using UBCG. GenBank accession numbers of the genomes used are given in parentheses. The gene support indices indicating the number of single gene trees supporting each branch in the tree from the concatenated alignment are marked on the branches. *Moraxella lacunata* NBRC 102154^T^ was used as outgroup. Bars, 0.20 substitutions per site.

The draft genomes of strains BSP-53ᵀ and BSP-153ᵀ were 3.58 Mb and 3.56 Mb in size, with DNA G+C contents of 41.67 mol% and 40.87 mol%, respectively, both falling within the known range for the genus *Acinetobacter* [38,39]. Genome assembly statistics, including completeness, contamination, contig counts and N50 values, are summarized in Table 1, and all genomes exhibited high completeness (>90%) and low contamination (<5%). Phylogenomic reconstruction based on 81 conserved single-copy core genes (UBCG pipeline) [40] robustly placed both strains within the genus *Acinetobacter* (bootstrap >80%; Fig. 1). Strain BSP-53ᵀ formed a stable, distinct lineage together with the unclassified strain *Acinetobacter* sp. ANC 4218, corresponding to the previously proposed *Acinetobacter* Taxon 32 [38]. Similarly, strain BSP-153ᵀ constituted a robust, distinct clade with four unclassified strains (ANC 4204, TUM 15064, SWAC57 and WCHAc060042), representing *Acinetobacter* Taxon 39 [38]. Both clades were clearly separated from all validly published type strains.

**Table 1.** Genomic features of strains BSP-53^T^ and BSP-153^T^, along with closely related strains within the genus *Acinetobacter*

Feature	BSP-53^T^	*Acinetobacter* sp. ANC 4218	*A. kookii* ANC 4667^T^	BSP-153^T^	*Acinetobacter* sp. ANC 4204	*Acinetobacter* sp. SWAC57	*Acinetobacter* sp. WCHAc060042	*Acinetobacter* sp. TUM15064	*Acinetobacter johnsonii* CIP 64.6^T^
Source of isolation	Soil	Clayey mud of a drained pond	Soil	Soil	Wetland water with mud	Hospital sewage	Sewage	Human sputum	Eviscerated chickens
Genome size (Mb)	3.58	3.39	3.09	3.56	3.90	4.11	3.82	3.81	3.61
G+C (%)	41.67	41.75	43.00	40.87	40.77	40.72	40.77	40.98	41.50
Total Scaf no.	153	35	22	47	19	50	157	240	11
Scaf N50 (bp)	47,750	197,333	288,148	156,392	429,441	209,966	80,550	49,267	1,088,563
Total Ctg no.	196	38	23	48	19	55	157	240	48
Ctg N50 (bp)	44,504	197,333	288,148	156,392	429,441	209,966	80,550	49,267	124,388
Completeness (%)	99.66	99.66	99.67	100	100	100	100	100	91.31
Contamination (%)	1.16	1.23	0.44	0.00	0.75	0.68	0.27	0.84	2.67
Total no. of genes	3,419	3,226	2,959	3,330	3,696	3,863	3,662	3,681	3,552

Genomic relatedness analysis further confirmed the distinct species status of BSP-53ᵀ and BSP-153ᵀ. GTDB-TK classified strain BSP-53ᵀ as *Acinetobacter* sp. 002135245, consistent with other strains in Taxon 32, and strain BSP-153ᵀ as *Acinetobacter* sp. 002165305, consistent with members of Taxon 39. This analysis systematically compared their genomes with (i) all 85 known *Acinetobacter* species with validly published and correct names and (ii) 2 unofficial taxa (Taxon 32 and Taxon 39), the latter comprising 7 additional strains (Tables S1 and S2). The ANIb and dDDH values between BSP-53ᵀ and *Acinetobacter* sp. ANC 4218 (Taxon 32) were 96.14 and 70.10%, respectively, confirming that they belong to the same species. Likewise, BSP-153ᵀ showed ANI values of 98.22–98.39% with its four conspecific strains within Taxon 39. In contrast, both proposed type strains exhibited significantly lower relatedness to their closest validly published relatives: BSP-53ᵀ versus *A. kookii* ANC 4667ᵀ (ANI 90.99%, dDDH 42.30%), and BSP-153ᵀ versus *A. johnsonii* CIP 64.6ᵀ (ANI 88.69%, dDDH 36.00%). All interspecies values were well below the accepted species thresholds (ANI 95–96%, dDDH 70%) [41,42], providing conclusive genomic evidence for their status as novel species.

Comparative genomic analysis revealed distinctive functional traits supporting their phylogenetic divergence. Strain BSP-53ᵀ showed a reduction in genes related to central metabolism compared to *A. kookii* ANC 4667ᵀ and *Acinetobacter* sp. ANC 4218, but an enhancement in polysaccharide biosynthesis. It also possessed numerous virulence-associated genes related to adhesion (56), nutrition/metabolism (50) and regulation (29). Strain BSP-153ᵀ had a smaller genome and fewer genes in most KEGG pathways than its conspecific strains (from Taxon 39) and *A. johnsonii* CIP 64.6ᵀ yet retained or expanded genes involved in xenobiotic biodegradation, glycan biosynthesis and terpenoid/polyketide metabolism. It also exhibited a larger repertoire of glycosyl transferases (26 genes) than related strains, suggesting enhanced polysaccharide synthesis capability. Virulence factor profiling showed that BSP-153ᵀ and its conspecific strains shared enrichment in genes involved in immune regulation, adherence and nutritional/metabolic functions compared to *A. johnsonii* CIP 64.6ᵀ. Analysis using the CARD database revealed that strains BSP-53^T^ and BSP-153^T^, along with their phylogenetic relatives within Taxa 32 and 39, harbour comparable intrinsic resistance determinants (e.g. against tetracyclines and fluoroquinolones, Tables S3 and S4) yet remain phenotypically susceptible *in vitro*. Importantly, unlike the clinically significant pathogen *A. baumannii*, these environmental lineages lack horizontally acquired high-risk elements such as specific carbapenemases, suggesting that they function as natural resistance reservoirs rather than posing acute clinical threats [12,14].

In summary, the phylogenomic, genomic relatedness and functional genomic data collectively satisfy the polyphasic criteria for the proposal of BSP-53ᵀ and BSP-153ᵀ as the type strains of two novel species within the genus *Acinetobacter*.

## Physiology and chemotaxonomy

Cell morphology was examined by transmission electron microscopy (JEM-1400; JEOL), and motility was assessed by light microscopy (Axiostar Plus, ZEISS). Gram staining was performed using a Gram-staining kit (Solarbio; catalogue no. G1060) according to the manufacturer’s instructions. The temperature range for growth was determined by incubating strains at 16, 25, 30, 37, 42 and 45 °C for 3 days. The pH range for growth was assessed in R2A medium adjusted to pH 4.0–9.0 (in increments of 0.5–1.0 pH units) during incubation at 30 °C for 3 days. NaCl tolerance was determined in liquid R2A medium supplemented with the addition of 0–5% (w/v) NaCl in 1% increments. Growth was monitored by measuring OD_600_ with a UV/visible spectrophotometer (SPECORD 205; Analytik Jena). Antibiotic susceptibility was tested by the disc diffusion method [43], measuring inhibition zone diameters. Biochemical and enzymatic activities were analysed with GEN III MicroPlates (Biolog), API 20 NE and API ZYM systems (bioMérieux) according to the manufacturers' protocols [44]. Whole-cell fatty acids and polar lipids were analysed as described previously [45].

The growth phenotypes of strains BSP-53^T^ and BSP-153^T^ were characterized with respect to temperature, pH and NaCl tolerance. Both strains grew in R2A medium at temperatures ranging from 16 to 42 °C but differed in their optimal growth temperatures. Strain BSP-53^T^ showed optimal growth at 30 °C, whereas its closest phylogenetic relative, *A. kookii* ANC 4667^T^, had an optimal growth temperature of 35 °C. Strain BSP-153^T^ exhibited optimal growth at 37 °C, differing from the 30 °C optimum of its reference strain *A. johnsonii* CIP 64.6^T^. Both strains grew in R2A medium at pH 5.0–9.0. The optimal pH was 6.5 for BSP-53^T^ and 7.5 for BSP-153^T^. NaCl tolerance tests showed that BSP-53^T^ tolerated 0–3% (w/v) NaCl, with an optimum in the absence of additional NaCl, whereas BSP-153^T^ tolerated 0–4% (w/v) NaCl, with an optimum at 1% (w/v) NaCl (Table 2).

**Table 2.** Differential characteristics of strains BSP-53^T^, BSP-153^T^, *A. kookii* ANC 4667^T^ and *A. johnsonii* CIP 64.6^T^ Strains: 1, BSP-53^T^; 2, BSP-153^T^; 3, *A. kookii* ANC 4667^T^; 4, *A. johnsonii* CIP 64.6^T^; +, positive; −, negative.

Characteristics	1	2	3	4
Source of isolation	Soil	Soil	Soil	Eviscerated chickens
Cell shape	Short rod-shaped	Short rod-shaped	Coccoid	Coccoid
Metabolic end product(s)				
Major cellular fatty acids	Summed feature 3 (C_16 : 1_* ω*7*c*, C_16 : 1_* ω*6*c*), C_18 : 1_* ω*9*c* and C_16 : 0_	Summed feature 3 (C_16 : 1_* ω*7*c*, C_16 : 1_* ω*6*c*), C_18 : 1_* ω*9*c* and C_16 : 0_	Summed feature 3 (C_16 : 1_* ω*7*c*, C_16 : 1_* ω*6*c*), C_18 : 1_* ω*9*c* and C_16 : 0_	Summed feature 3 (C_16 : 1_* ω*7*c*, C_16 : 1_* ω*6*c*), C_18 : 1_* ω*9*c* and C_16 : 0_
Major polar lipid	DPG, PE, PG and PL	DPG, PE and PG	DPG, PE, PG and UPL	DPG, PE, PG, UPL, UAPL and UAL1
Growth conditions (optimal):				
Temperature (°C)	16–42 (30)	16–42 (37)	24–41 (35)	15–37 (30)
pH	5.0–9.0 (6.5)	5.0–9.0 (7.5)	6.0–9.0 (7)	6.0–9.0 (7)
Acidification of d-glucose	−	+	−	−
Haemolysis of sheep blood	−	−	−	−
Utilization of:				
Acetate	+	+	+	+
4-Aminobutyrate	+	+	+	−
l-Alanine	+	+	+	−
l-Arginine	+	+	−	+
l-Aspartate	−	−	−	−
Bromo-succinate	+	+	−	−
Citrate	−	+	−	−
l-Glutamate	+	+	−	−
l-Lactate	+	+	+	+
l-Malate	+	+	−	+

**Fig. 2.**
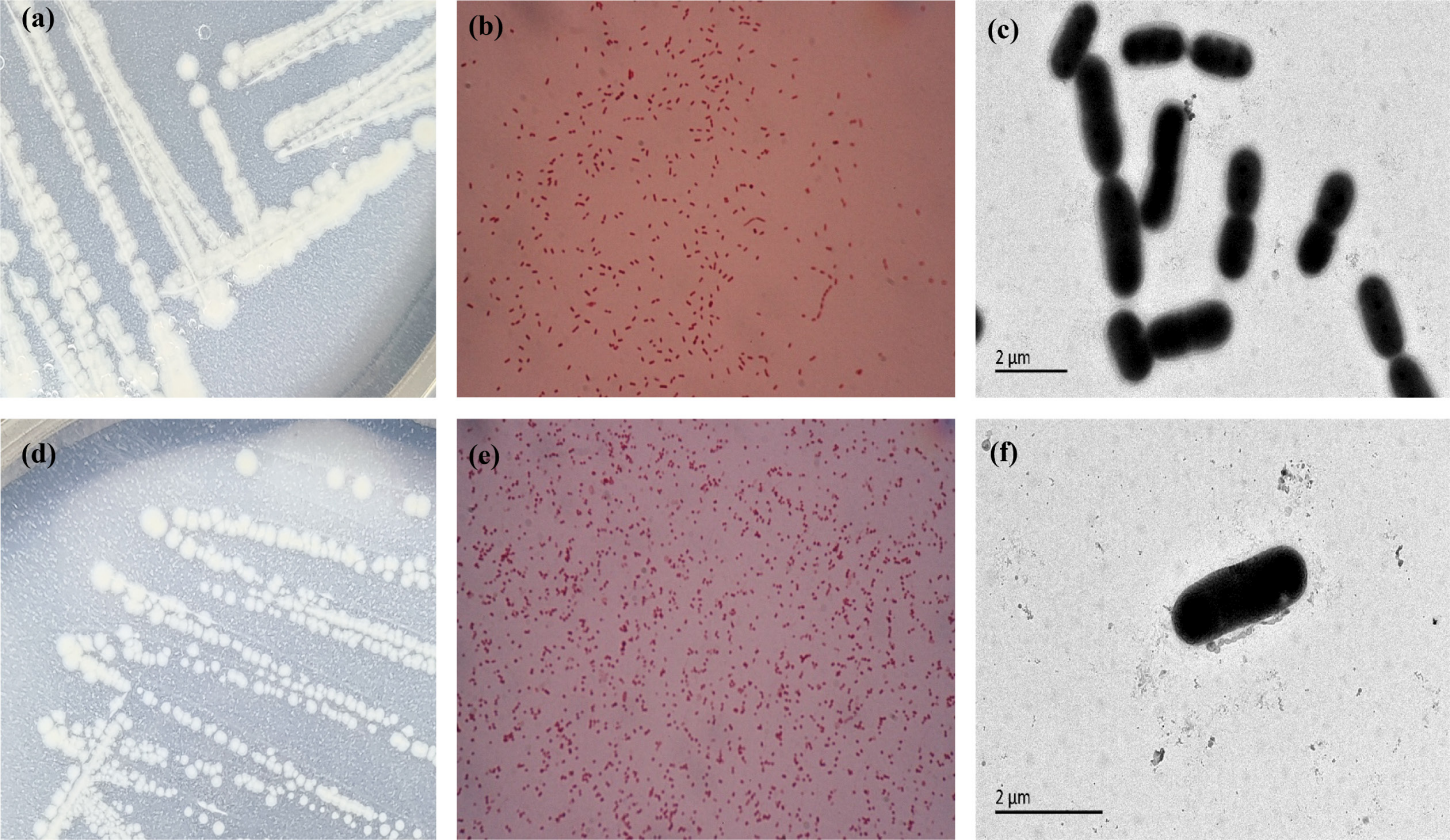
Cell and colony morphology of strains BSP-53^T^ and BSP-153^T^. (**a, b**) Cell morphology of strains BSP-53^T^ and BSP-153^T^ on R2A solid medium; (**c, d**) BSP-53^T^ and BSP-153^T^ Gram-stained cells; (**e, f**) BSP-53^T^ and BSP-153^T^ transmission microscopy.

Phenotypic characterization, including biochemical properties, polar lipids and cellular fatty acids, was performed under optimal growth conditions. Both strains formed round, creamy-white, opaque colonies with moist, slightly raised surfaces on R2A agar. Colonies of BSP-53^T^ measured 2–3 mm with irregular margins (Fig. 2a), whereas those of BSP-153^T^ measured 1–2 mm in diameter with smooth margins (Fig. 2b). Transmission electron microscopy revealed that both strains were non-flagellated and exhibited a short rod-shaped morphology (Fig. 2e, f). Cellular morphology differed between BSP-53^T^ and the reference strain *A. kookii* ANC 4667^T^: BSP-53^T^ was short rod-shaped, while *A. kookii* ANC 4667^T^ was coccoid. This morphological distinction supports their taxonomic separation. Similarly, BSP-153^T^ displayed a short rod-shaped morphology, contrasting with the coccoid morphology of its reference strain *A. johnsonii* CIP 64.6^T^. Gram staining confirmed that all strains were Gram-negative (Fig. 2c, d). Strains BSP-53^T^ and BSP-153^T^ were sensitive to multiple *β*-lactams, tetracyclines and fluoroquinolones, but resistant to oxacillin, lincomycin, vancomycin, polymyxin B and clindamycin. In addition, strain BSP-153^T^ exhibited resistance to cefazolin and azithromycin (Fig. S5). These susceptibility profiles were generally consistent with their resistome patterns (Table S3). However, both strains remained phenotypically susceptible to tetracyclines and fluoroquinolones despite harbouring the corresponding resistance genes. These genotype–phenotype discrepancies suggest that intrinsic resistance mechanisms or uncharacterized determinants may contribute to the observed resistance profiles.

Substrate utilization profiles of strains BSP-53^T^ and BSP-153^T^ were determined using GEN III MicroPlates (Biolog), API ZYM and API 20 NE systems. As summarized in Table 2, strain BSP-53^T^ utilized acetate, 4-aminobutyrate, l-alanine, l-arginine, bromo-succinate, l-glutamate, l-lactate and l-malate, whereas its phylogenetic relative *A. kookii* ANC 4667^T^ only utilized acetate, 4-aminobutyrate, l-alanine and l-lactate. Similarly, strain BSP-153^T^ utilized acetate, 4-aminobutyrate, l-alanine, l-arginine, l-aspartate, citrate, l-glutamate, l-lactate and l-malate, while its reference strain *A. johnsonii* CIP 64.6^T^ utilized only acetate, l-arginine, l-lactate and l-malate. These differential substrate assimilation patterns provide valuable phenotypic characteristics for distinguishing the novel strains from their closest related species (Table S5). Enzymatic activity profiles determined with API ZYM and API 20 NE systems showed that BSP-53^T^ exhibited weak *α*-galactosidase and *β*-galactosidase activities in addition to alkaline phosphatase and esterase activities but was negative for *β*-glucuronidase, *α*-mannosidase, *α*-fucosidase, starch hydrolysis, gelatin hydrolysis, urease and d-glucose acidification. In contrast, BSP-153^T^ showed *β*-galactosidase, *α*-glucosidase and *β*-glucuronidase activities; was negative for *α*-mannosidase and *α*-fucosidase; and tested positive for d-glucose acidification. This represents a distinct phenotypic difference between the two strains. Both strains were negative for oxidase, nitrate reductase, indole production, gelatin liquefaction, *β*-galactosidase (ONPG) and urease activity. These shared characteristics provide valuable taxonomic information (Tables S5, S6 and S7).

Strains BSP-53^T^ and BSP-153^T^ shared similar major fatty acids (>10%) with other members of the genus *Acinetobacter* [38,39], which were summed feature 3 (C_16 : 1_* ω7*c*/*C1_6 : 1_* ω6*c), C_18 : 1_* ω9*c and C_16 : 0_. However, strain BSP-53^T^ could be distinguished from * A. kookii* ANC 4667^T^ by its higher amount of C_16 : 0_ and lower amount of summed feature 8 (C_18 : 1_* ω7*c*/*ω*6*c). Similarly, strain BSP-153^T^ differed from *A. johnsonii* CIP 64.6^T^ in its higher amounts of C_18 : 1_* ω9*c and C_16 : 0_ and a lower amount of summed feature 3 (Table 3). The polar lipid profiles of the strains were also determined. Strain BSP-53^T^ contained the major polar lipids diphosphatidylglycerol (DPG), phosphatidylethanolamine (PE) and phosphatidylglycerol (PG), which are common in the genus *Acinetobacter* [38,39]. In contrast, *A. kookii* ANC 4667^T^ additionally contained two unidentified aminolipids and one unidentified aminophospholipid. Strain BSP-153^T^ also possessed DPG, PE and PG but lacked two unidentified lipids present in *A. johnsonii* CIP 64.6^T^. These polar lipid profiles, together with the fatty acid compositions, support the distinction of strains BSP-53^T^ and BSP-153^T^ from their closely related species (Fig. S4).

**Table 3.** Cellular fatty acid profiles (% of total) of strains BSP-53^T^, BSP-153^T^, *A. kookii* ANC 4667^T^ and *A. johnsonii* CIP 64.6^T^

Fatty acid	BSP-53^T^	BSP-153^T^	*A. kookii* ANC 4667^T^	*A. johnsonii* CIP 64.6^T^
C_18 : 1_* ω*9*c*	26.7	37.1	21.7	29.7
C_16 : 0_	24.0	23.6	13.1	13.3
C_12 : 0_	8.5	8.5	7.8	7.5
C_12 : 0_ 3-OH	5.8	5.6	3.4	3.4
Summed feature 3^*^	26.9	15.7	24.8	20.0
Summed feature 8^*^	3.9	3.4	6.3	3.1

*Summed feature 3 consists of C_16 : 1_* ω*7*c*/C_16 : 1_* ω*6*c* and C_16 : 1_* ω*6*c*/C_16 : 1_* ω*7*c*; summed feature 8 consists of C_18 : 1_* ω*7*c* and C_18 : 1_* ω*6*c*.

These chemotaxonomic profiles confirm the strains' phylogenetic placement within the genus *Acinetobacter* through conserved features (summed feature 3, DPG-PE-PG core), while the strain-specific variations in C_16 : 0_/_C18 : 1_* ω9*c ratios and accessory lipid components (PL/UPL) provide diagnostic markers supporting their recognition as two distinct novel species.

Integrating phylogenetic, phenotypic and chemotaxonomic evidence, we propose that strains BSP-53^T^ and BSP-153^T^ represent two novel species within the genus *Acinetobacter*, with the respective names *Acinetobacter uliginosus* sp. nov. and *Acinetobacter halobius* sp. nov.

## Description of *Acinetobacter uliginosus* sp. nov.

*Acinetobacter uliginosus* sp. nov. (u.li.gi.no’sus. L. masc. adj. *uliginosus,* marshy.)

Cells are Gram-stain-negative, aerobic, non-spore-forming, non-motile and short rod-shaped. After 3-day incubation on R2A medium at 30 °C, colonies are 2–3 mm in diameter, circular, creamy-white and opaque, with a moist, slightly convex surface and irregular margins. Growth occurs at 16–42 °C (optimum, 30 °C), at pH 5.0–9.0 (optimum, pH 6.5) and in the presence of 0–3% (w/v) NaCl (optimum, 0%). Negative for oxidase activity, starch hydrolysis, d-glucose acidification, sheep blood haemolysis, nitrate reduction, gelatin liquefaction and urease activity. The major cellular fatty acids comprise summed feature 3 (comprising C_16 : 1_* ω*7*c*/C_16 : 1_* ω*6*c*), C_18 : 1_* ω*9*c*, C_16 : 0_, C_12 : 0_ and C_12 : 0_ 3-OH. The predominant polar lipids are DPG, PE, PG and an unidentified phospholipid (PL).

The genomic DNA G+C content of the type strain is 41.67 mol%. The type strain, BSP-53ᵀ (=CGMCC 1.62070ᵀ=KCTC 8190ᵀ), was isolated from a soil sample collected from the Longxing Aquatic System in Beijing Olympic Park, China. The 16S rRNA gene sequence has been deposited in GenBank/EMBL/DDBJ under accession number OR140835. The complete genome sequence has been submitted under accession number JBIQOC000000000 (version JBIQOC000000000).

## Description of *Acinetobacter halobius* sp. nov.

*Acinetobacter halobius* sp. nov. (ha.lo’bi.us. Gr. masc. n. *hals*, salt; Gr. masc. n. *bios*, life; N.L. masc. adj. *halobius*, salt-living, referring to the optimal growth at 1% NaCl concentration exhibited by the type strain).

Cells are Gram-stain-negative, aerobic, non-spore-forming, non-motile and short rod-shaped. After 3-day incubation on R2A medium at 37 °C, colonies are 1–2 mm in diameter, circular, creamy-white, opaque, with a moist, slightly convex surface and smooth margins. Growth occurs at 16–42 °C (optimum, 37 °C), at pH 5.0–9.0 (optimum, pH 7.5) and in the presence of 0–4% (w/v) NaCl (optimum, 1%). Positive for d-glucose acidification. Negative for oxidase activity, starch hydrolysis, sheep blood haemolysis, nitrate reduction, gelatin liquefaction and urease activity. The major cellular fatty acids comprise C_18 : 1_* ω*9*c*, C_16 : 0_, summed feature 3 (C_16 : 1_* ω*7*c*/C_16 : 1_* ω*6*c*), C_12 : 0_ and C_12 : 0_ 3-OH. The predominant polar lipids are DPG, PE and PG.

The genomic DNA G+C content of the type strain is 40.87 mol%. The type strain, BSP-153ᵀ (=CGMCC 1.62071ᵀ=KCTC 8191ᵀ), was isolated from a soil sample collected from the Longxing Aquatic System in Beijing Olympic Park, China. The 16S rRNA gene sequence has been deposited in GenBank/EMBL/DDBJ under accession number OR140844. The complete genome sequence has been submitted under accession number JBIQOD000000000 (version JBIQOD000000000).

## Supplementary material

10.1099/ijsem.0.007192 Supplementary Material 1.

## Abbreviations

ANI: average nucleotide identity
CGMCC: China General Microbiological Culture Collection Centre
dDDH: digital DNA–DNA hybridization
DPG: diphosphatidylglycerol
GTDB-TK: Genome Taxonomy Database Toolkit
KCTC: Korean Collection for Type Cultures
ML: maximum-likelihood
MP: maximum parsimony
NJ: neighbor-joining
PE: phosphatidylethanolamine
PG: phosphatidylglycerol
PL: phospholipid
R2A: Reasoner's 2A
