# Supplementary material for: Acinetobacter uliginosus sp. nov. and Acinetobacter halobius sp. nov. isolated from soil
Source: Int J Syst Evol Microbiol. 2026 Jun 4;76(6):007192. doi: 10.1099/ijsem.0.007192 (PMC13237956; doi:10.1099/ijsem.0.007192)
Supplement: Supplementary Material 1. [file ijsem-76-07192-s001.pdf]

Acinetobacter uliginosus sp. nov. and *Acinetobacter halobius* sp. nov. isolated from soil

Cong-Guo Ran[1,2], Feng-Lan Liu[4], Tong Wu[1], Rashidin Abdugheni[3], Nan Zhou[1*], Shuang-Jiang Liu[1,2,4*]

Author affiliations:

[1] State Key Laboratory of Microbial Diversity and Innovative Utilization, and Environmental Microbiology Research Center at Institute of Microbiology, Chinese Academy of Sciences, Beijing, 100101, P. R. China.

[2] University of the Chinese Academy of Sciences, Beijing, 100049, P. R. China.

[3]Department of Microbiology, School of Basic Medical Sciences, Xinjiang Medical University, Urumqi 830017, China.

[4] State Key Laboratory of Microbial Biotechnology, Shandong University, Qingdao, 266237, P. R. China.

*Correspondence: Shuang-Jiang Liu (liusj@im.ac.cn) and Nan Zhou (joel.c@126.com)
Institute of Microbiology, Chinese Academy of Sciences
No.1 Beichen West Road, Chaoyang District, Beijing 100101, China
Tel: +86-10-64807423; Fax: +86-10-64807421

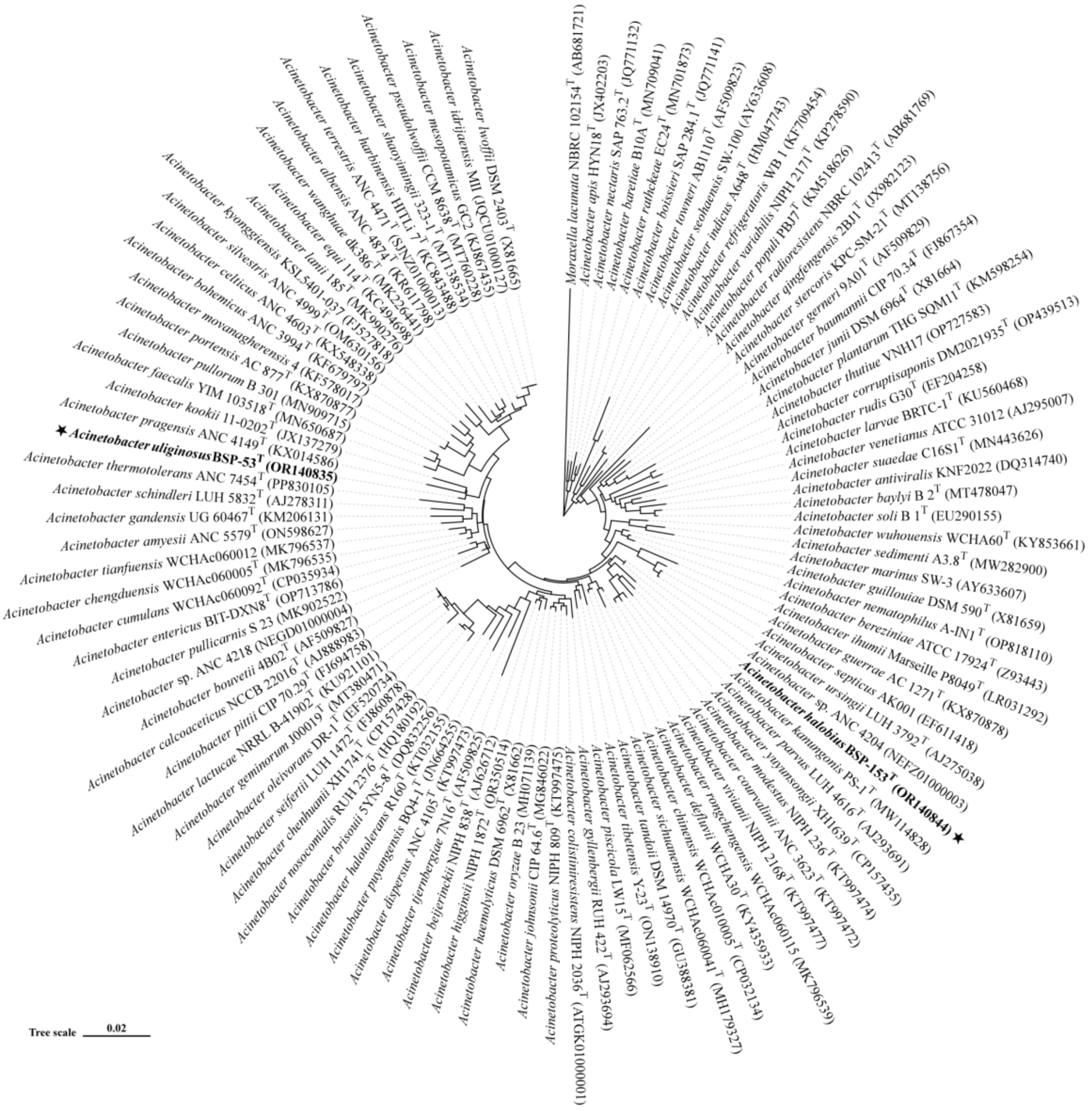

Figure S1. Neighbor-joining tree based on 16S rRNA gene sequences showing the phylogenetic position of strains BSP-53^{T} and BSP-153^{T} among the members of genus *Acinetobacter*. *Moraxella lacunata* NBRC 102154^{T} served as the out-group. The sequences were obtained from GenBank. Bar, 0.01 substitutions per nucleotide position.

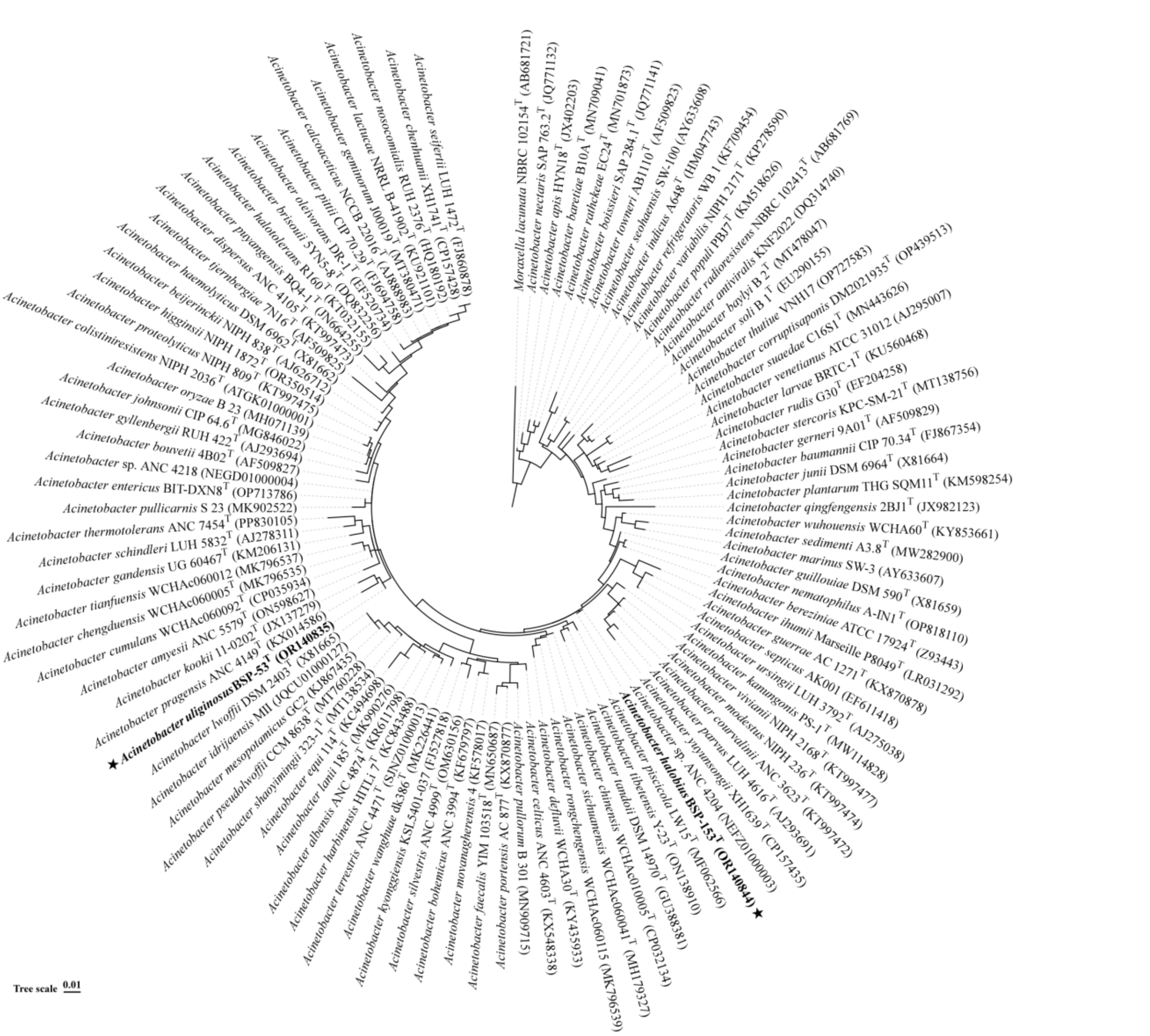

Figure S2. Maximum-likelihood tree based on 16S rRNA gene sequences showing the phylogenetic position of strains BSP-53^T and BSP-153^T among the members of genus *Acinetobacter*. *Moraxella lacunata* NBRC 102154^T served as the out-group. The sequences were obtained from GenBank. Bar, 0.01 substitutions per nucleotide position.

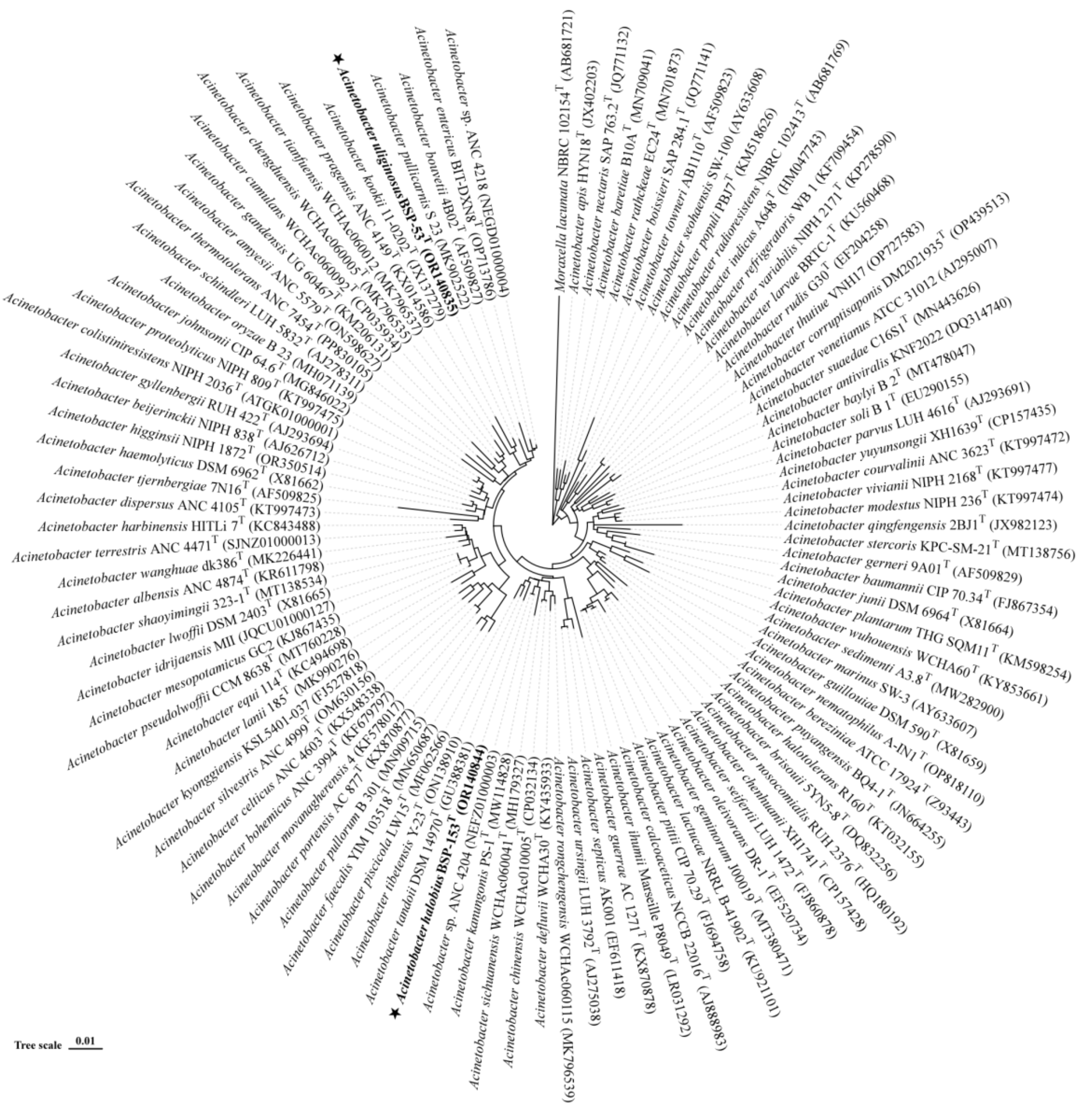

Figure S3. Maximum-parsimony tree based on 16S rRNA gene sequences showing the phylogenetic position of strains BSP-53^{T} and BSP-153^{T} among the members of genus *Acinetobacter*. *Moraxella lacunata* NBRC 102154^{T} served as the out-group. The sequences were obtained from GenBank. Bar, 0.01 substitutions per nucleotide position.

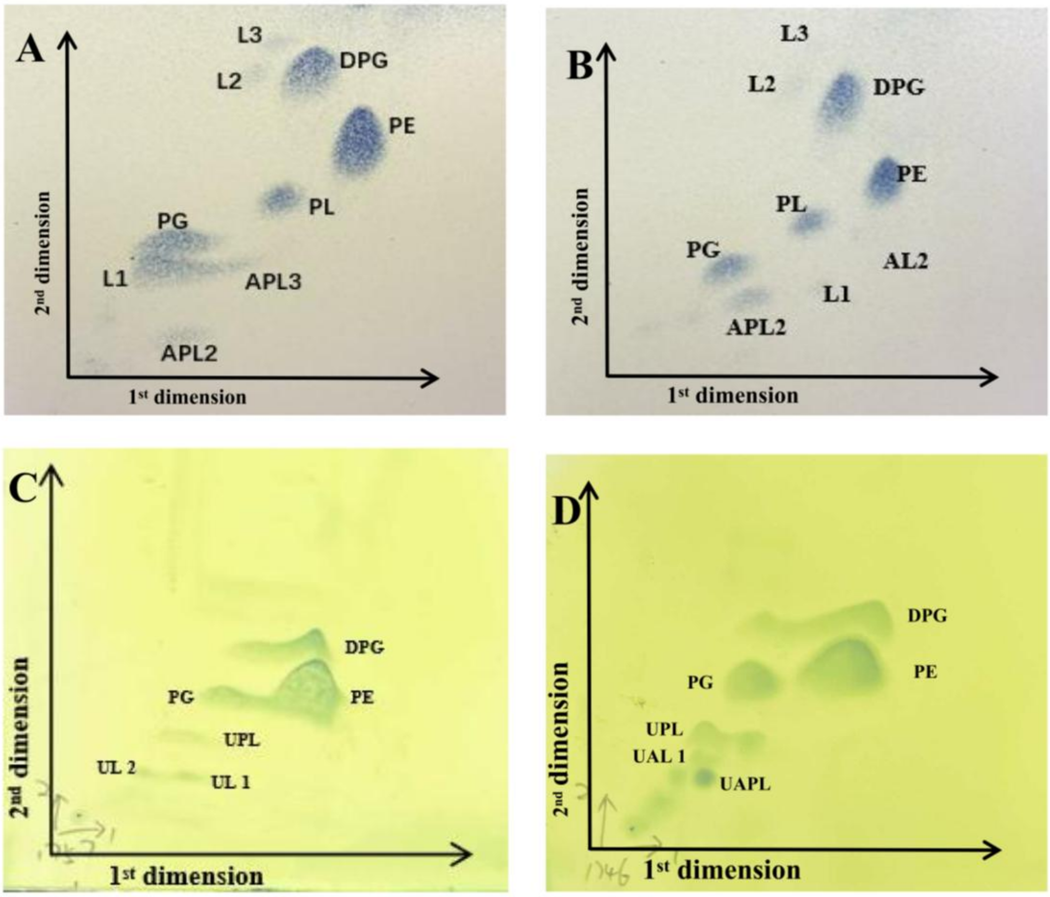

Figure S4. Polar lipids of strains BSP-53^{T} and BSP-153^{T} with type strains *A. johnsonii* CIP 64.6^{T} and *A. kookii* ANC 4667^{T}. (A and B), strains (A) BSP-53^{T} and (B) BSP-153^{T} Polar lipids; (C and D), type strains (C) *A. johnsonii* CIP 64.6^{T} and (D) *A. kookii* ANC 4667^{T} Polar lipids; DPG: diphosphatidylglycerol; PG: phosphatidylglycerol; PE: phosphatidylethanolamine; UPL: unidentified phospholipid; UAL1-2: unidentified aminolipids; UAPL: unidentified aminophospholipid; UL1-2: unidentified lipids.

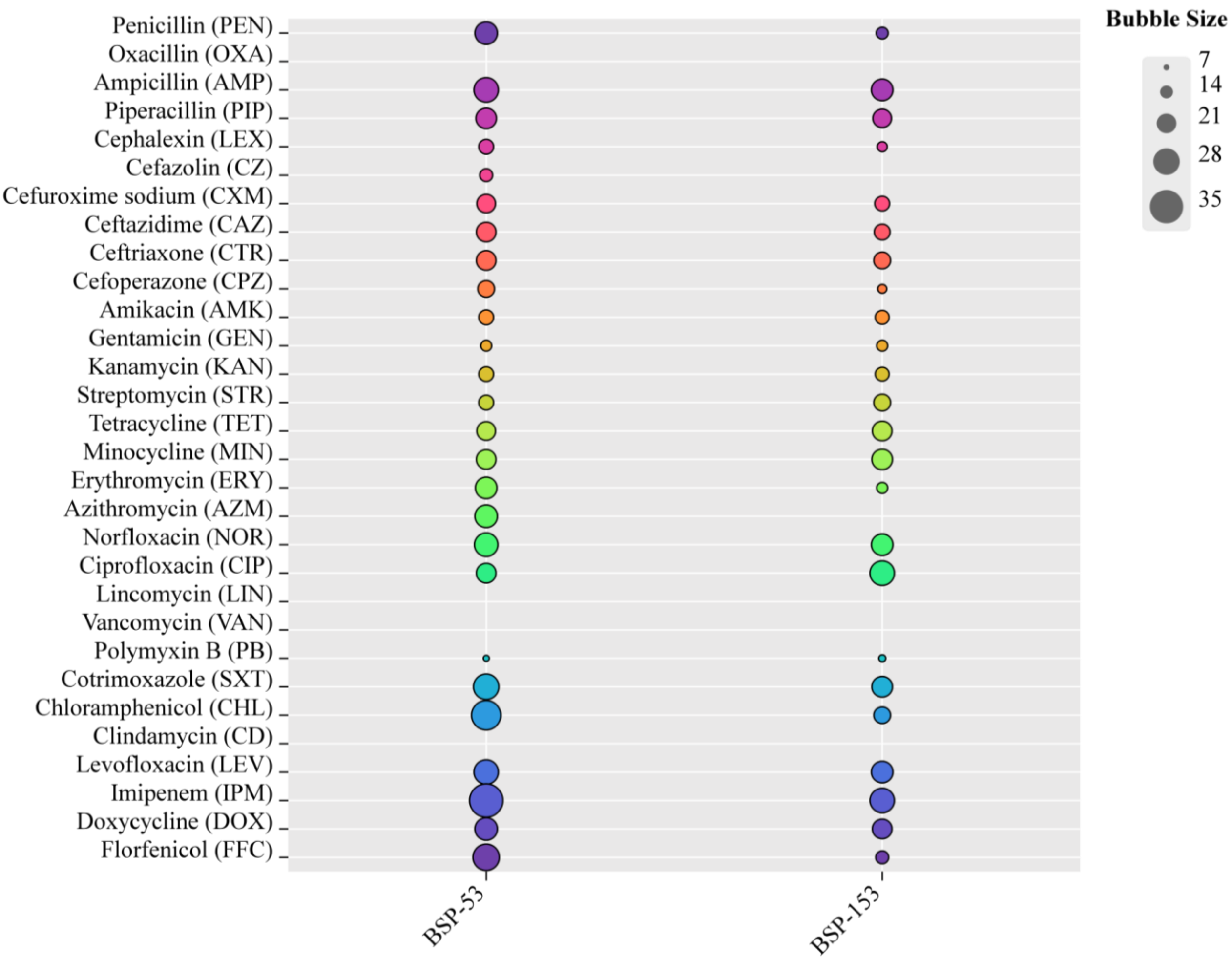

Figure S5. Antibiotic inhibition zones of strains BSP-53^T and BSP-153^T. The bubble size indicates the size of the inhibition zone (mm).

Table S1. Digital DNA–DNA hybridization (dDDH) and average nucleotide identity (ANIb) values between *Acinetobacter* sp. strain BSP-53^{T} and the type strains of closely related species, including *Acinetobacter* sp. Taxon 32.

Name	Strain	GCA no.	dDDH	ANI
Acinetobacter sp.	ANC 4218	GCA_002135245.1	70.10	96.30
Acinetobacter kookii	ANC 4667^{T}	GCA_900096895.1	42.30	90.71
Acinetobacter sp.	ANC 4169	GCA_002135355.1	31.70	86.18
Acinetobacter terrae	ANC 4282^{T}	GCA_013004375.1	25.80	82.14
Acinetobacter terrestris	ANC 4471^{T}	GCA_004331155.1	25.40	81.79
Acinetobacter bohemicus	ANC 3994^{T}	GCA_000367925.1	23.90	80.15
Acinetobacter albensis	ANC 4874^{T}	GCA_900095025.1	21.90	78.51
Acinetobacter harbinensis	HITLi7^{T}	GCA_000816495.1	21.80	78.11
Acinetobacter variabilis	NIPH 2171^{T}	GCA_000369625.1	23.30	77.69
Acinetobacter lwoffii	DSM 2403^{T}	GCA_029024105.1	22.70	77.53
Acinetobacter towneri	DSM 14962^{T}	GCA_000368785.1	22.70	77.51
Acinetobacter indicus	TQ23^{T}	GCA_009914475.1	22.10	77.38
Acinetobacter thermotolerans	ANC 7454^{T}	GCA_039867205.1	23.00	77.36
Acinetobacter pseudolwoffii	CCM 8638^{T}	GCA_042647985.1	22.20	77.21
Acinetobacter entericus	BIT-DXN8^{T}	GCA_026168575.1	22.80	77.21
Acinetobacter johnsonii	CIP 64.6^{T}	GCA_000368045.1	22.50	76.98
Acinetobacter tandoii	DSM 14970^{T}	GCA_000621065.1	22.70	76.92
Acinetobacter schindleri	CIP 107287^{T}	GCA_000368625.1	22.10	76.74
Acinetobacter gandensis	ANC 4275^{T}	GCA_001678755.1	21.70	76.74
Acinetobacter celticus	ANC 4603^{T}	GCA_001707755.1	21.00	76.68
Acinetobacter amyesii	ANC 5579^{T}	GCA_023499985.1	22.10	76.58
Acinetobacter faecalis	YIM 103518^{T}	GCA_009707625.1	21.80	76.47
Acinetobacter chengduensis	WCHAc060005^{T}	GCA_003664645.1	22.40	76.46
Acinetobacter bouvetii	DSM 14964^{T}	GCA_000368865.1	21.30	76.40
Acinetobacter cumulans	WCHAc060092^{T}	GCA_003024525.3	22.30	76.39
Acinetobacter chinensis	WCHAc010005^{T}	GCA_002165375.2	21.80	76.39
Acinetobacter tibetensis	Y-23^{T}	GCA_023824315.1	21.30	76.33
Acinetobacter zhairhuonensis	A7.4^{T}	GCA_047300875.1	21.30	76.28
Acinetobacter pragensis	ANC 4149^{T}	GCA_001605895.1	21.20	76.25

Acinetobacter kanungonis	PS-1^{T}	GCA_009939195.1	21.00	76.19
Acinetobacter lanii	185^{T}	GCA_011578285.1	22.30	76.12
Acinetobacter wanghuae	dk386^{T}	GCA_009557235.1	21.00	76.12
Acinetobacter wuhouensis	WCHA60^{T}	GCA_001696605.3	22.60	76.06
Acinetobacter shaoyimingii	323-1^{T}	GCA_011578045.1	22.10	75.68
Acinetobacter equi	114^{T}	GCA_001307195.1	21.00	75.59
Acinetobacter silvestris	ANC 4999^{T}	GCA_002135235.1	21.00	75.52
Acinetobacter piscicola	LW15^{T}	GCA_002233755.1	21.40	75.51
Acinetobacter defluvii	WCHA30^{T}	GCA_001704615.3	22.70	75.51
Acinetobacter gerneri	DSM 14967^{T}	GCA_000368565.1	24.20	75.50
Acinetobacter sichuanensis	WCHAc060041^{T}	GCA_003024515.2	21.90	75.21
Acinetobacter guillouiae	NBRC 110550^{T}	GCA_002370525.2	21.50	75.12
Acinetobacter bereziniae	GD03185^{T}	GCA_016576965.1	22.00	74.99
Acinetobacter radioresistens	NBRC 102413^{T}	GCA_006757745.1	21.50	74.91
Acinetobacter ursingii	DSM 16037^{T}	GCA_000368825.1	22.30	74.86
Acinetobacter parvus	DSM 16617^{T}	GCA_000368025.1	22.80	74.73
Acinetobacter corruptisaponis	KCTC 92772^{T}	GCA_030053775.1	22.60	74.72
Acinetobacter nematophilus	A-IN1^{T}	GCA_026344175.1	20.80	74.67
Acinetobacter colistiniresistens	NIPH 2036^{T}	GCA_000413935.1	21.90	74.57
Acinetobacter portensis	AC877^{T}	GCA_009372215.1	21.90	74.57
Acinetobacter haemolyticus	CIP 64.3^{T}	GCA_000369065.1	22.50	74.40
Acinetobacter stercoris	KPC-SM-21^{T}	GCA_900323515.1	21.10	74.35
Acinetobacter courvalinii	CCM 8635^{T}	GCA_014635545.1	20.80	74.27
Acinetobacter nosocomialis	XH1679^{T}	GCA_041021905.1	21.30	74.16
Acinetobacter higginsii	NIPH 1869^{T}	GCA_022549575.1	21.60	74.16
Acinetobacter brisouii	CIP 110357^{T}	GCA_000488275.1	21.80	74.11
Acinetobacter proteolyticus	NIPH 809^{T}	GCA_000367945.1	21.00	74.11
Acinetobacter vivianii	NIPH 2168^{T}	GCA_000369705.1	20.70	74.11
Acinetobacter geminorum	J00019^{T}	GCA_013009345.1	20.90	74.08
Acinetobacter dispersus	ANC 4105^{T}	GCA_000369485.1	20.60	74.07
Acinetobacter modestus	NIPH 236^{T}	GCA_000367965.1	21.60	74.06
Acinetobacter baumannii	CIP 70.34^{T}	GCA_019331655.1	20.40	74.01
Acinetobacter ihumii	MarseilleP8049^{T}	GCA_900625095.1	20.30	73.94
Acinetobacter oleivorans	DR1^{T}	GCA_000196795.1	20.40	73.87

Acinetobacter pittii	CIP 70.29^{T}	GCA_000369045.1	20.20	73.85
Acinetobacter tjernbergiae	DSM 14971^{T}	GCA_000759995.1	20.40	73.83
Acinetobacter calcoaceticus	CIP 81.8^{T}	GCA_000368965.1	20.40	73.80
Acinetobacter gyllenbergii	FMP01^{T}	GCA_001682515.1	20.60	73.80
Acinetobacter seifertii	S21^{T}	GCA_016064815.1	20.60	73.79
Acinetobacter lactucae	NRRLB 41902^{T}	GCA_001605885.1	20.10	73.74
Acinetobacter guerrae	AC1271^{T}	GCA_009372255.1	19.70	73.73
Acinetobacter suaedae	C16S1^{T}	GCA_008630915.1	20.60	73.61
Acinetobacter beijerinckii	CIP 110307^{T}	GCA_000369005.1	20.80	73.59
Acinetobacter halotolerans	JCM 31009^{T}	GCA_004208515.1	20.40	73.56
Acinetobacter baylyi	ADP1^{T}	GCA_000046845.1	20.20	73.54
Acinetobacter junii	CIP 64.5^{T}	GCA_000368765.1	22.10	73.49
Acinetobacter soli	CIP 110264^{T}	GCA_000368705.1	19.90	73.33
Acinetobacter rudis	CIP 110305^{T}	GCA_000413895.1	21.70	73.12
Acinetobacter larvae	BRTC 1^{T}	GCA_001704115.1	20.90	72.65
Acinetobacter populi	PBJ7^{T}	GCA_002174125.1	21.10	71.89
Acinetobacter sedimenti	A3.8^{T}	GCA_022601685.1	23.20	71.82
Acinetobacter puyangensis	JCM 18011^{T}	GCA_039542245.1	20.50	71.39
Acinetobacter qingfengensis	CCUG 69710^{T}	GCA_008693185.1	20.70	71.06
Acinetobacter nectaris	CIP 110549^{T}	GCA_000488215.1	20.30	71.05
Acinetobacter rathckeae	EC24^{T}	GCA_015627125.1	20.40	71.01
Acinetobacter apis	ANC 5114^{T}	GCA_900197575.1	19.50	70.97
Acinetobacter boissieri	ANC 4422^{T}	GCA_900096955.1	20.10	70.81
Acinetobacter baretiae	B10A^{T}	GCA_015627105.1	21.30	70.62

Table S2. Digital DNA–DNA hybridization (dDDH) and average nucleotide identity (ANIb) values between *Acinetobacter* sp. strain BSP-153^{T} and the type strains of closely related species, including *Acinetobacter* sp. Taxon 39.

Name	Strain	GCA no.	dDDH	ANI
Acinetobacter sp.	ANC 4204	GCA_002135205.1	85.90	98.26
Acinetobacter sp.	SWAC57	GCA_003569905.1	85.90	98.18
Acinetobacter sp.	TUM 15064	GCA_008980265.1	85.60	98.07
Acinetobacter sp.	WCHAc060042	GCA_003231195.1	85.00	98.14

Acinetobacter sp.	WCHA55	GCA_002165305.2	39.50	89.47
Acinetobacter johnsonii	CIP 64.6^T	GCA_000368045.1	36.00	88.26
Acinetobacter sedimenti	A3.8^T	GCA_022601685.1	23.00	71.87
Acinetobacter tibetensis	Y-23^T	GCA_023824315.1	22.90	77.44
Acinetobacter entericus	BIT-DXN8^T	GCA_026168575.1	22.20	75.93
Acinetobacter tandoii	DSM 14970^T	GCA_000621065.1	22.20	76.71
Acinetobacter wuhouensis	WCHA60^T	GCA_001696605.3	22.10	75.47
Acinetobacter shaoyimingii	323-1^T	GCA_011578045.1	21.90	75.42
Acinetobacter towneri	DSM 14962^T	GCA_000368785.1	21.90	76.88
Acinetobacter cumulans	WCHAc060092^T	GCA_003024525.3	21.80	76.12
Acinetobacter lanii	185^T	GCA_011578285.1	21.70	75.53
Acinetobacter parvus	DSM 16617^T	GCA_000368025.1	21.70	74.15
Acinetobacter brisouii	CIP 110357^T	GCA_000488275.1	21.60	73.73
Acinetobacter modestus	NIPH 236^T	GCA_000367965.1	21.60	74.04
Acinetobacter zhairhuonensis	A7.4^T	GCA_047300875.1	21.60	76.30
Acinetobacter amyesii	ANC 5579^T	GCA_023499985.1	21.50	76.12
Acinetobacter bohemicus	ANC 3994^T	GCA_000367925.1	21.40	76.50
Acinetobacter chengduensis	WCHAc060005^T	GCA_003664645.1	21.40	75.70
Acinetobacter faecalis	YIM103518^T	GCA_009707625.1	21.40	76.02
Acinetobacter gandensis	ANC 4275^T	GCA_001678755.1	21.40	76.03
Acinetobacter gerneri	DSM 14967^T	GCA_000368565.1	21.40	74.61
Acinetobacter bouvetii	DSM 14964^T	GCA_000368865.1	21.30	75.58
Acinetobacter equi	114^T	GCA_001307195.1	21.30	75.65
Acinetobacter terrae	ANC 4282^T	GCA_013004375.1	21.30	76.35
Acinetobacter bereziniae	GD03185^T	GCA_016576965.1	21.20	74.92
Acinetobacter defluvii	WCHA30^T	GCA_001704615.3	21.20	75.25
Acinetobacter guillouiae	NBRC 110550^T	GCA_002370525.2	21.20	74.96
Acinetobacter lwoffii	DSM 2403^T	GCA_029024105.1	21.20	75.56
Acinetobacter piscicola	LW15^T	GCA_002233755.1	21.20	75.07
Acinetobacter pseudolwoffii	CCM 8638^T	GCA_042647985.1	21.20	75.50
Acinetobacter chinensis	WCHAc010005^T	GCA_002165375.2	21.10	74.99
Acinetobacter dispersus	ANC 4105^T	GCA_000369485.1	21.10	74.07
Acinetobacter kanungonis	PS-1^T	GCA_009939195.1	21.10	75.91
Acinetobacter pragensis	ANC 4149^T	GCA_001605895.1	21.10	75.27

Acinetobacter sichuanensis	WCHAc060041^T	GCA_003024515.2	21.10	74.99
Acinetobacter variabilis	NIPH 2171^T	GCA_000369625.1	21.10	75.41
Acinetobacter albensis	ANC 4874^T	GCA_900095025.1	21.00	75.94
Acinetobacter beijerinckii	CIP 110307^T	GCA_000369005.1	21.00	73.74
Acinetobacter boissieri	ANC 4422^T	GCA_900096955.1	21.00	71.02
Acinetobacter corruptisaponis	KCTC 92772^T	GCA_030053775.1	21.00	73.56
Acinetobacter courvalinii	CCM 8635^T	GCA_014635545.1	21.00	73.41
Acinetobacter larvae	BRTC1^T	GCA_001704115.1	21.00	72.68
Acinetobacter terrestris	ANC 4471^T	GCA_004331155.1	21.00	76.31
Acinetobacter tjernbergiae	DSM 14971^T	GCA_000759995.1	21.00	74.10
Acinetobacter wanghuae	dk386^T	GCA_009557235.1	21.00	75.65
Acinetobacter celticus	ANC 4603^T	GCA_001707755.1	20.90	76.17
Acinetobacter colistiniresistens	NIPH 2036^T	GCA_000413935.1	20.90	73.87
Acinetobacter junii	CIP 64.5^T	GCA_000368765.1	20.90	73.57
Acinetobacter kookii	ANC 4667^T	GCA_900096895.1	20.90	76.23
Acinetobacter portensis	AC877^T	GCA_009372215.1	20.90	73.87
Acinetobacter haemolyticus	CIP 64.3^T	GCA_000369065.1	20.80	73.82
Acinetobacter rudis	CIP 110305^T	GCA_000413895.1	20.80	72.90
Acinetobacter vivianii	NIPH 2168^T	GCA_000369705.1	20.80	73.84
Acinetobacter baretiae	B10A^T	GCA_015627105.1	20.70	71.02
Acinetobacter halotolerans	JCM 31009^T	GCA_004208515.1	20.70	73.56
Acinetobacter indicus	TQ23^T	GCA_009914475.1	20.70	75.36
Acinetobacter proteolyticus	NIPH 809^T	GCA_000367945.1	20.70	73.99
Acinetobacter qingfengensis	CCUG 69710^T	GCA_008693185.1	20.70	70.85
Acinetobacter rathckeae	EC24^T	GCA_015627125.1	20.70	71.22
Acinetobacter schindleri	CIP 107287^T	GCA_000368625.1	20.70	74.97
Acinetobacter thermotolerans	ANC 7454^T	GCA_039867205.1	20.70	75.30
Acinetobacter higginsii	NIPH 1869^T	GCA_022549575.1	20.60	73.76
Acinetobacter nematophilus	A-IN1^T	GCA_026344175.1	20.60	74.20
Acinetobacter nosocomialis	XH1679^T	GCA_041021905.1	20.60	73.82
Acinetobacter pittii	CIP 70.29^T	GCA_000369045.1	20.60	73.72
Acinetobacter populi	PBJ7^T	GCA_002174125.1	20.60	71.30
Acinetobacter seifertii	S21^T	GCA_016064815.1	20.60	73.81
Acinetobacter suaedae	C16S1^T	GCA_008630915.1	20.60	73.65

Acinetobacter baumannii	CIP 70.34^{T}	GCA_019331655.1	20.50	73.72
Acinetobacter calcoaceticus	CIP 81.8^{T}	GCA_000368965.1	20.50	73.75
Acinetobacter geminorum	J00019^{T}	GCA_013009345.1	20.50	73.65
Acinetobacter gyllenbergii	FMP 01^{T}	GCA_001682515.1	20.50	73.61
Acinetobacter nectaris	CIP 110549^{T}	GCA_000488215.1	20.50	71.50
Acinetobacter oleivorans	DR1^{T}	GCA_000196795.1	20.50	73.70
Acinetobacter ursingii	DSM 16037^{T}	GCA_000368825.1	20.50	73.61
Acinetobacter silvestris	ANC 4999^{T}	GCA_002135235.1	20.40	74.97
Acinetobacter lactucae	NRRLB 41902^{T}	GCA_001605885.1	20.30	73.49
Acinetobacter stercoris	KPC-SM-21^{T}	GCA_900323515.1	20.30	73.82
Acinetobacter harbinensis	HITLi7^{T}	GCA_000816495.1	20.20	75.17
Acinetobacter puyangensis	JCM 18011^{T}	GCA_039542245.1	20.20	71.00
Acinetobacter baylyi	ADP1^{T}	GCA_000046845.1	20.00	73.25
Acinetobacter soli	CIP 110264^{T}	GCA_000368705.1	20.00	72.88
Acinetobacter radioresistens	NBRC 102413^{T}	GCA_006757745.1	19.90	73.26
Acinetobacter apis	ANC 5114^{T}	GCA_900197575.1	19.80	71.13
Acinetobacter guerrae	AC1271^{T}	GCA_009372255.1	19.80	73.32
Acinetobacter ihumii	MarseilleP8049^{T}	GCA_900625095.1	19.80	73.36

Table S3. Genomic analysis of strains BSP-53^{T} and BSP-153^{T}, along with closely related strains within the genus *Acinetobacter*.

		BSP-53^{T}	*A. kookii* ANC 4667^{T}	*Acinetobacter* sp. ANC 4218	*Acinetobacter* sp. ANC 4169	BSP-153^{T}	*A. johnsonii* CIP 64.6^{T}	*Acinetobacter* sp. WCHA55	*Acinetobacter* sp. ANC 4204	*Acinetobacter* sp. TUM 15064	*Acinetobacter* sp. WCHAc060042	*Acinetobacter* sp. SWAC57
KEGG	metabolism	2935	3107	3090	3191	2998	3226	3388	3601	3567	3463	3489
	cellular processes	129	175	160	184	140	166	170	193	204	200	194
	genetic information processing	212	224	221	205	202	215	216	217	216	221	219
	organismal systems	72	78	83	75	80	88	89	97	104	94	98
	human diseases	145	167	166	169	176	199	209	201	223	199	212
	environmental information processing	180	218	213	223	201	227	233	259	262	259	263
	total	3673	3969	3933	4047	3797	4121	4305	4568	4576	4436	4475
CAZy	glycosyl transferases （GTs）	23	29	25	23	26	16	16	19	17	21	19
	carbohydrate esterases (CEs）	19	15	15	17	19	18	18	18	21	18	19
	glycoside hydrolases (GHs）	12	9	12	11	11	9	15	16	13	11	13
	auxiliary activities (AAs）	9	10	8	11	11	9	12	9	12	11	11
	carbohydrate-binding modules (CBMs）	1	1	1	1	0	0	1	1	1	1	0
	total	64	64	61	63	67	52	62	63	64	62	62
VFDB	immune regulation	67	67	63	61	71	56	67	68	67	69	71

	adherence	56	48	52	51	58	48	48	49	51	49	53
	effector delivery system	34	46	33	50	41	52	41	46	49	48	51
	nutritional/metabolic factor	50	40	41	42	86	54	66	82	85	77	77
	exotoxins	18	20	21	22	21	23	25	28	25	27	27
	regulation	29	19	22	17	22	18	22	21	20	22	18
	biofilm	20	15	21	17	21	12	15	20	22	17	17
	stress survival	14	13	13	14	13	15	16	13	15	13	16
	motility	14	22	20	20	17	16	19	24	24	23	22
	other	21	22	25	22	26	26	33	35	34	35	33
	total	323	312	311	316	376	320	352	386	392	380	385
CARD*	macrolide antibiotics	31	30	28	26	33	26	31	27	31	29	31
	tetracycline antibiotics	35	40	35	35	49	37	48	51	56	49	49
	peptide antibiotics	34	31	33	27	30	31	34	37	40	43	36
	fluoroquinolone antibiotics	28	37	37	34	34	33	34	44	46	38	36
	penam	26	31	22	24	33	27	38	34	36	35	35
	cephalosporins	24	29	22	24	32	26	36	32	33	32	33
	disinfectants and anti-infectives	26	26	27	29	29	26	27	28	34	32	30
	carbapenems	16	25	14	22	24	22	31	22	24	23	23
	phenicol antibiotic	20	18	17	17	30	19	26	24	24	22	21

other	162	154	152	155	179	177	204	181	190	186	185
total	402	421	387	393	473	424	509	480	514	489	479

*Note: The relatively high counts observed in certain antibiotic classes (e.g., carbapenems) primarily reflect the presence of intrinsic multidrug efflux systems rather than acquired resistance determinants. Manual curation confirmed the absence of horizontally transferred high-risk elements, such as specialized carbapenemases or extended-spectrum *β*-lactamases (ESBLs).

Table S4. The number of annotated genes of KEGG metabolism pathways of strains BSP-53^{T} and BSP-153^{T}, along with closely related strains within the genus *Acinetobacter*.

	BSP-53^{T}	*A. kookii* ANC 4667^{T}	*Acinetobacter* sp. ANC 4218	*Acinetobacter* sp. ANC 4169	BSP-153^{T}	*A. johnsonii* CIP 64.6^{T}	*Acinetobacter* sp. WCHA55	*Acinetobacter* sp. ANC 4204	*Acinetobacter* sp. TUM 15064	*Acinetobacter* sp. WCHAc060042	*Acinetobacter* sp. SWAC57
**Biosynthesis of other secondary metabolites**	44	46	47	57	45	60	57	56	63	55	56
**Global and overview maps**	1563	1654	1659	1704	1607	1732	1824	1938	1930	1865	1880
**Xenobiotics biodegradation and metabolism**	131	125	121	169	147	133	160	204	176	167	171
**Carbohydrate metabolism**	264	309	283	291	259	292	304	308	306	303	303
**Lipid metabolism**	88	111	103	117	93	125	134	130	122	139	131
**Metabolism of cofactors and vitamins**	160	180	184	176	159	185	200	205	204	205	200
**Amino acid metabolism**	282	280	283	286	284	287	291	323	318	296	308
**Glycan biosynthesis and metabolism**	59	55	54	51	61	49	56	56	58	60	56
**Nucleotide metabolism**	78	77	84	80	81	87	90	98	98	94	93
**Metabolism of other amino acids**	55	52	54	53	52	61	62	62	62	60	63
**Metabolism of terpenoids and polyketides**	43	39	36	40	47	42	38	38	39	40	38
**Energy metabolism**	168	179	182	167	163	173	172	183	191	179	190

Table S5. Phenotypic characteristics of strains BSP-53^T and BSP-153^T determined by Biolog GEN III MicroPlate™.

"+": Positive; "-": Negative

NO.	Characteristic	BSP-53^T	BSP-153^T	*A. kookii* ANC 4667^T	*A. johnsonii* CIP 64.6^T
A1	Negative control	–	–	–	–
A2	Dextrin	–	–	–	–
A3	D-Maltose	–	–	–	–
A4	D-Trehalose	–	–	–	–
A5	D-Cellobiose	–	–	–	–
A6	Gentiobiose	–	–	–	–
A7	Sucrose	–	–	–	–
A8	D-Turanose	–	–	–	–
A9	Stachyose	–	–	–	–
B1	D-Raffinose	–	–	–	–
B2	α-D-Lactose	–	–	–	–
B3	D-Melibiose	–	–	–	–
B4	β-Methyl-D-Glucoside	–	–	–	–
B5	D-Salicin	–	–	–	–
B6	N-Acetyl-D-Glucosamine	–	–	–	–
B7	N-Acetyl-β-D-Mannosamine	–	–	–	–
B8	N-Acetyl-D-Galactosamine	–	–	–	–
B9	N-Acetyl-Neuraminic acid	–	–	–	–
C1	α-D-Glucose	–	–	–	–
C2	D-Mannose	–	–	–	–
C3	D-Fructose	–	–	–	–
C4	D-Galactose	–	–	–	–
C5	3-Methyl glucose	–	–	–	–
C6	D-Fucose	–	–	–	–
C7	L-Fucose	–	–	–	–
C8	L-Rhamnose	–	–	–	–
C9	Inosine	–	–	–	–
D1	D-Sorbitol	–	–	–	–
D2	D-Mannitol	–	–	–	–
D3	D-Arabitol	–	–	–	–
D4	myo-Inositol	–	–	–	–
D5	Glycerol	–	–	–	–
D6	D-Glucose-6-PO4	–	–	–	–
D7	D-Fructose-6-PO4	–	–	–	–
D8	D-Aspartic Acid	–	–	–	–

D9	D-Serine	−	−	−	−
E1	Gelatin	−	−	−	−
E2	Glycyl-L-Proline	−	−	−	−
E3	L-Alanine	+	+	+	−
E4	L-Arginine	+	+	−	+
E5	L-Aspartic acid	+	+	−	−
E6	L-Glutamic acid	+	+	−	−
E7	L-Histidine	−	+	−	+
E8	L-Pyroglutamic acid	+	+	−	−
E9	L-Serine	−	−	−	−
F1	Pectin	−	−	−	+
F2	D-Galacturonic acid	−	−	−	−
F3	L-Galactonic acid lactone	−	+	−	+
F4	D-Gluconic acid	−	−	−	−
F5	D-Glucuronic acid	−	−	−	−
F6	Glucuronamide	−	+	+	−
F7	Mucic acid	+	−	−	−
F8	Quinic acid	−	−	−	−
F9	D-Saccharic acid	−	−	−	−
G1	p-Hydroxy-Phenylacetic acid	+	−	−	−
G2	Methyl pyruvate	+	+	+	−
G3	D-Lactic acid methyl ester	+	+	+	−
G4	L-Lactic acid	+	+	+	−
G5	Citric acid	+	+	−	−
G6	α-Keto-Glutaric acid	+	−	+	+
G7	D-Malic acid	+	−	−	+
G8	L-Malic acid	+	+	−	+
G9	Bromo-Succinic acid	+	+	−	−
H1	Tween 40	+	+	+	−
H2	γ-Amino-Butyric acid	+	+	+	−
H3	α-Hydroxy-Butyric acid	+	+	+	−
H4	β-Hydroxy-D,L-Butyric acid	+	+	−	−
H5	α-Keto-Butyric acid	+	+	+	−
H6	Acetoacetic acid	+	−	−	−
H7	Propionic acid	+	+	+	+
H8	Acetic acid	+	+	+	+
H9	Formic acid	−	−	−	−
A10	Positive control	+	+	+	+
A11	pH 6	+	+	+	+
A12	pH 5	+	+	−	−
B10	1% NaCl	+	+	+	+
B11	4% NaCl	−	+	−	−

B12	8% NaCl	−	−	−	−
C10	1% Sodium lactate	+	+	+	+
C11	Fusidic acid	−	−	−	−
C12	D-Serine	+	+	+	+
D10	Troleandomycin	−	+	+	−
D11	Rifamycin SV	+	+	+	+
D12	Minocycline	−	−	−	−
E10	Lincomycin	+	+	+	+
E11	Guanidine HCl	+	+	+	−
E12	Niaproof 4	+	+	−	+
F10	Vancomycin	+	+	+	+
F11	Tetrazolium violet	+	+	−	+
F12	Tetrazolium blue	+	+	−	+
G10	Nalidixic acid	−	+	+	−
G11	Lithium chloride	+	+	−	−
G12	Potassium tellurite	+	+	+	+
H10	Aztreonam	+	+	+	+
H11	Sodium butyrate	+	+	+	+
H12	Sodium bromate	+	−	−	−

Table S6. Phenotypic characteristics of strains BSP-53^T and BSP-153^T determined by the API ZYM kit.

“+”: Positive; “-”: Negative

NO.	Characteristic	BSP-53^T	BSP-153^T
1	Control	−	−
2	Alkaline phosphatase	+	+
3	Esterase (C4)	+	+
4	Esterase (C8)	−	−
5	Lipase (C14)	−	−
6	Leucine aminopeptidase	−	−
7	Valine aminopeptidase	−	−
8	Cysteine aminopeptidase	−	−
9	Trypsin	−	−
10	Chymotrypsin	−	−
11	Acid phosphatase	+	+
12	Phosphoamidase	−	−
13	α-Galactosidase	−	−
14	β-Galactosidase	−	−

15	*β*-Glucuronidase	−	−
16	*α*-Glucosidase	−	−
17	*β*-Glucosidase	−	−
18	*N*-acetyl-*β*-glucosaminidase	−	−
19	*α*-mannosidase	−	−
20	*α*-fucosidase	−	−

Table S7. Phenotypic characteristics of strains BSP-53^{T} and BSP-153^{T} determined by the API 20NE test kit.

"+": Positive; "-": Negative

NO.	Characteristic	BSP-53^{T}	BSP-153^{T}
1	Reduction of nitrates to nitrites	−	−
2	Reduction of nitrates to nitrogen	−	−
3	Indole production (tryptophan)	−	−
4	Fermentation (glucose)	−	+
5	Arginine dihydrolase	−	−
6	Urease	−	−
7	Hydrolysis (*β*-glucosidase) (esculin)	−	−
8	Hydrolysis (protease) (gelatin)	−	−
9	*β*-Galactosidase	−	−
10	Assimilation (glucose)	−	−
11	Assimilation (arabinose)	−	−
12	Assimilation (mannose)	−	−
13	Assimilation (mannitol)	−	−
14	Assimilation (N-acetyl-glucosamine)	−	−
15	Assimilation (maltose)	−	−
16	Assimilation (potassium gluconate)	−	−
17	Assimilation (capric acid)	+	+
18	Assimilation (adipic acid)	−	−
19	Assimilation (malate)	+	+
20	Assimilation (trisodium citrate)	−	−
21	Assimilation (phenylacetic acid)	−	−
22	Cytochrome oxidase	−	−
